# Supplementary material for: Istaroxime for Patients with Acute Heart Failure: A Systematic Review and Meta-Analysis of Randomized Controlled Trials
Source: Diseases. 2023 Dec 17;11(4):183. doi: 10.3390/diseases11040183 (PMC10743119; doi:10.3390/diseases11040183)
Supplement: Supplementary file 1 [file diseases-11-00183-s001.zip › diseases-2741649-supplementary.pdf]

diseases

Systematic Review

Istaroxime for Patients with Acute Heart Failure: A Systematic Review and Meta-Analysis of Randomized Controlled Trials

Mohamed Abuelazm [1,†], Shafaqat Ali [2,†], Majd M. AlBarakat [3], Abdelrahman Mahmoud [4], Mohammad Tanashat [5], Husam Abu Suilik [6], Basel Abdelazeem [7] and James Robert Brašić [8,9,10,*]

1 Faculty of Medicine, Tanta University, Tanta 31527, Egypt; dr.mabuelazm@gmail.com
2 Department of Internal Medicine, Louisiana State University Health Shreveport, Shreveport, LA 71103, USA; shafaqat231@gmail.com
3 Faculty of Medicine, Jordan University of Science and Technology, Irbid 22110, Jordan; mmalbarakat20@med.just.edu.jo
4 Faculty of Medicine, Minia University, Minia 61519, Egypt; abdelrahman.mahmoud20211997@gmail.com
5 Faculty of Medicine, Yarmouk University, Irbid 21163, Jordan; mohammad_tanashat@hotmail.com
6 Faculty of Medicine, Hashemite University, Zarqa 13133, Jordan; husamabusuilik.md@gmail.com
7 Division of Cardiology, Department of Medicine, West Virginia University School of Medicine, Morgantown, WV 26506, USA; baselelramly@gmail.com
8 Section of High-Resolution Brain Positron Emission Tomography Imaging, Division of Nuclear Medicine and Molecular Imaging, The Russell H. Morgan Department of Radiology and Radiological Science, The Johns Hopkins University School of Medicine, Baltimore, MD 21287, USA
9 Department of Psychiatry, New York City Health and Hospitals/Bellevue, New York, NY 10016, USA
10 Department of Psychiatry, New York University Grossman School of Medicine, New York University Langone Health, New York, NY 10016, USA
* Correspondence: jbrasic1@jh.edu; Tel.: +1-410-986-0341
† These authors contributed equally to this work.

Supplementary Materials:

Content:

Tables:

Table S1. Search strategy.

Table S2. Risk of bias assessment for Carubelli et al. 2020 [20].

Table S3. Risk of bias assessment for Metre et al. 2022 [19].

Table S4. Risk of bias assessment for Shah et al. 2009 [21].

Table S5. Sensitivity analysis.

Figures:

Figure S1. Forest plot of the safety outcomes.

Table S1. Search Strategy.

Database	Search Terms	Search Field	Search Results
Pubmed	(Istaroxime OR PST 2744 OR PST2744 OR PST-2744) AND ("heart failure" OR "cardiac failure" OR "heart decompensation" OR HFrEF OR HFpEF)	All Field	51
Cochrane	(Istaroxime OR PST 2744 OR PST2744 OR PST-2744) AND ("heart failure" OR "cardiac failure" OR "heart decompensation" OR HFrEF OR HFpEF)	All Field	18
WOS	(Istaroxime OR PST 2744 OR PST2744 OR PST-2744) AND ("heart failure" OR "cardiac failure" OR "heart decompensation" OR HFrEF OR HFpEF)	All Field	69
SCOPUS	TITLE-ABS-KEY ((istaroxime OR pst 2744 OR pst2744 OR pst-2744) AND ("heart failure" OR "cardiac failure" OR "heart decompensation" OR hfref OR hfpef))	Title, Abstract, Keywords	15
EMBASE	#3. #1 AND #2<br>#2. 'heart failure':ti,ab,kw OR 'cardiacfailure':ti,ab,kw OR 'heart decompensation':ti,ab,kw OR hfref:ti,ab,kw OR hfpef:ti,ab,kw<br>#1. istaroxime:ti,ab,kw OR pst2744:ti,ab,kw OR 'pst':ti,ab,kw	All Field	63

Table S2. Risk of bias assessment for Carubelli et al. 2020 [20].

Domain	Signalling question	Response
**Bias arising from the randomization process**	1.1 Was the allocation sequence random?	Y
	1.2 Was the allocation sequence concealed until participants were enrolled and assigned to interventions?	Y
	1.3 Did baseline differences between intervention groups suggest a problem with the randomization process?	N
	**Risk of bias judgement**	**Low**
**Bias due to deviations from intended interventions**	2.1.Were participants aware of their assigned intervention during the trial?	N
	2.2.Were carers and people delivering the interventions aware of participants' assigned intervention during the trial?	NI
	2.3. If Y/PY/NI to 2.1 or 2.2: Were there deviations from the intended intervention that arose because of the experimental context?	PN
	2.4 If Y/PY to 2.3: Were these deviations likely to have affected the outcome?	NA
	2.5. If Y/PY/NI to 2.4: Were these deviations from intended intervention balanced between groups?	NA
	2.6 Was an appropriate analysis used to estimate the effect of assignment to intervention?	PY
	2.7 If N/PN/NI to 2.6: Was there potential for a substantial impact (on the result) of the failure to analyse participants in the group to which they were randomized?	NA
	**Risk of bias judgement**	**Low**
**Bias due to missing outcome data**	3.1 Were data for this outcome available for all, or nearly all, participants randomized?	Y
	3.2 If N/PN/NI to 3.1: Is there evidence that result was not biased by missing outcome data?	NA
	3.3 If N/PN to 3.2: Could missingness in the outcome depend on its true value?	NA
	3.4 If Y/PY/NI to 3.3: Is it likely that missingness in the outcome depended on its true value?	NA
	**Risk of bias judgement**	**Low**
**Bias in measurement of the outcome**	4.1 Was the method of measuring the outcome inappropriate?	N
	4.2 Could measurement or ascertainment of the outcome have differed between intervention groups?	PN
	4.3 Were outcome assessors aware of the intervention received by study participants?	NI
	4.4 If Y/PY/NI to 4.3: Could assessment of the outcome have been influenced by knowledge of intervention received?	PN
	4.5 If Y/PY/NI to 4.4: Is it likely that assessment of the outcome was influenced by knowledge of intervention received?	NA
	**Risk of bias judgement**	**Low**

**Bias in selection of the reported result**	5.1 Were the data that produced this result analysed in accordance with a pre-specified analysis plan that was finalized before unblinded outcome data were available for analysis?	PY
	5.2 ... multiple eligible outcome measurements (e.g. scales, definitions, time points) within the outcome domain?	N
	5.3 ... multiple eligible analyses of the data?	N
	**Risk of bias judgement**	**Low**
**Overall bias**	**Risk of bias judgement**	**Low**

Table S3. Risk of bias assessment for Metre et al. 2022 [19].

Domain	Signalling question	Response
**Bias arising from the randomization process**	1.1 Was the allocation sequence random?	Y
	1.2 Was the allocation sequence concealed until participants were enrolled and assigned to interventions?	Y
	1.3 Did baseline differences between intervention groups suggest a problem with the randomization process?	PN
	**Risk of bias judgement**	**Low**
**Bias due to deviations from intended interventions**	2.1.Were participants aware of their assigned intervention during the trial?	N
	2.2.Were carers and people delivering the interventions aware of participants' assigned intervention during the trial?	PN
	2.3. If Y/PY/NI to 2.1 or 2.2: Were there deviations from the intended intervention that arose because of the experimental context?	NA
	2.4 If Y/PY to 2.3: Were these deviations likely to have affected the outcome?	NA
	2.5. If Y/PY/NI to 2.4: Were these deviations from intended intervention balanced between groups?	NA
	2.6 Was an appropriate analysis used to estimate the effect of assignment to intervention?	PY
	2.7 If N/PN/NI to 2.6: Was there potential for a substantial impact (on the result) of the failure to analyse participants in the group to which they were randomized?	NA
	**Risk of bias judgement**	**Low**
**Bias due to missing outcome data**	3.1 Were data for this outcome available for all, or nearly all, participants randomized?	Y
	3.2 If N/PN/NI to 3.1: Is there evidence that result was not biased by missing outcome data?	NA
	3.3 If N/PN to 3.2: Could missingness in the outcome depend on its true value?	NA
	3.4 If Y/PY/NI to 3.3: Is it likely that missingness in the outcome depended on its true value?	NA
	**Risk of bias judgement**	**Low**
**Bias in measurement of the outcome**	4.1 Was the method of measuring the outcome inappropriate?	PN
	4.2 Could measurement or ascertainment of the outcome have differed between intervention groups?	PN
	4.3 Were outcome assessors aware of the intervention received by study participants?	PN
	4.4 If Y/PY/NI to 4.3: Could assessment of the outcome have been influenced by knowledge of intervention received?	NA
	4.5 If Y/PY/NI to 4.4: Is it likely that assessment of the outcome was influenced by knowledge of intervention received?	NA
	**Risk of bias judgement**	**Low**

**Bias in selection of the reported result**	5.1 Were the data that produced this result analysed in accordance with a pre-specified analysis plan that was finalized before unblinded outcome data were available for analysis?	PY
	5.2 ... multiple eligible outcome measurements (e.g. scales, definitions, time points) within the outcome domain?	PN
	5.3 ... multiple eligible analyses of the data?	PN
	**Risk of bias judgement**	**Low**
**Overall bias**	**Risk of bias judgement**	**Low**

Table S4. Risk of bias assessment for Shah et al. 2009 [21].

Domain	Signalling question	Response
**Bias arising from the randomization process**	1.1 Was the allocation sequence random?	Y
	1.2 Was the allocation sequence concealed until participants were enrolled and assigned to interventions?	Y
	1.3 Did baseline differences between intervention groups suggest a problem with the randomization process?	N
	**Risk of bias judgement**	**Low**
**Bias due to deviations from intended interventions**	2.1.Were participants aware of their assigned intervention during the trial?	PN
	2.2.Were carers and people delivering the interventions aware of participants' assigned intervention during the trial?	NI
	2.3. If Y/PY/NI to 2.1 or 2.2: Were there deviations from the intended intervention that arose because of the experimental context?	PN
	2.4 If Y/PY to 2.3: Were these deviations likely to have affected the outcome?	NA
	2.5. If Y/PY/NI to 2.4: Were these deviations from intended intervention balanced between groups?	NA
	2.6 Was an appropriate analysis used to estimate the effect of assignment to intervention?	PY
	2.7 If N/PN/NI to 2.6: Was there potential for a substantial impact (on the result) of the failure to analyse participants in the group to which they were randomized?	NA
	**Risk of bias judgement**	**Low**
**Bias due to missing outcome data**	3.1 Were data for this outcome available for all, or nearly all, participants randomized?	PY
	3.2 If N/PN/NI to 3.1: Is there evidence that result was not biased by missing outcome data?	NA
	3.3 If N/PN to 3.2: Could missingness in the outcome depend on its true value?	NA
	3.4 If Y/PY/NI to 3.3: Is it likely that missingness in the outcome depended on its true value?	NA
	**Risk of bias judgement**	**Low**
**Bias in measurement of the outcome**	4.1 Was the method of measuring the outcome inappropriate?	PN
	4.2 Could measurement or ascertainment of the outcome have differed between intervention groups?	PN
	4.3 Were outcome assessors aware of the intervention received by study participants?	NI
	4.4 If Y/PY/NI to 4.3: Could assessment of the outcome have been influenced by knowledge of intervention received?	PN
	4.5 If Y/PY/NI to 4.4: Is it likely that assessment of the outcome was influenced by knowledge of intervention received?	NA
	**Risk of bias judgement**	**Low**

**Bias in selection of the reported result**	5.1 Were the data that produced this result analysed in accordance with a pre-specified analysis plan that was finalized before unblinded outcome data were available for analysis?	PY
	5.2 ... multiple eligible outcome measurements (e.g. scales, definitions, time points) within the outcome domain?	PN
	5.3 ... multiple eligible analyses of the data?	PN
	**Risk of bias judgement**	**Low**
**Overall bias**	**Risk of bias judgement**	**Low**

Table S5. Sensitivity analysis.

Outcome	Number of participants (Istaromime/Placebo)	Number of trials	Quantitative data synthesis				Heterogeneity analysis		
			MD	95% CI	Z value	*p* value	df	*p* value	I2 (%)
**LV end-diastolic volume**									
All studies	189/99	4	-4.69	[-12.85, 3.48]	1.12	0.26	3	0.00001	92
Omitting Carubelli et al. 2020 [20] (Cohort 1)	151/80	3	-6.21	[-17.05, 4.64]	1.12	0.26	2	0.0001	91
Omitting Carubelli et al. 2020 [20] (Cohort 2)	156/81	3	-7.10	[-16.29, 2.09]	1.52	0.13	2	0.00001	93
Omitting Metre et al. 2022 [19] (SEISMiC)	160/68	3	-0.49	[-4.42, 3.45]	0.24	0.81	2	0.24	29
Omitting Shah et al. 2009 [21] (HORIZON-HF)	100/68	3	-3.74	[-13.11, 5.63]	.78	0.43	2	0.00001	95
**LV end-systolic volume**									
All studies	189/99	4	-5.40	[-12.05, 1.25]	1.59	0.11	3	0.00001	91
Omitting Carubelli et al. 2020 [20] (Cohort 1)	151/80	3	-6.32	[-15.22, 2.57]	1.39	0.16	2	0.0002	88
Omitting Carubelli et al. 2020 [20] (Cohort 2)	156/81	3	-7.28	[-14.60, 0.03]	1.95	0.05	2	0.0001	91
Omitting Metre et al. 2022 [19] (SEISMiC)	160/68	3	-2.38	[-5.04, 0.27]	1.75	0.08	2	0.42	0
Omitting Shah et al. 2009 [21] (HORIZON-HF)	100/68	3	-5.10	[-12.86, 2.65]	1.29	0.20	2	0.00001	94
**E/e ratio**									
All studies	99/66	3	-1.04	[-4.15, 2.07]	0.65	0.51	2	0.00001	93
Carubelli et al. 2020 [20] (Cohort 1)	62/49	2	-0.20	[-3.73, 3.33]	0.11	0.91	1	0.00001	95
Omitting Carubelli et al. 2020 [20] (Cohort 2)	66/48	2	-0.57	[-4.99, 3.85]	0.25	0.80	1	0.0004	92
Omitting Metre et al. 2022 [19] (SEISMiC)	70/35	2	-2.33	[-3.64, -1.03]	3.50	0.0005	1	0.54	0

**IVC diameter**									
All studies	110/70	3	-1.82	[-3.74, 0.10]	1.86	0.06	2	0.0007	86
Omitting Carubelli et al. 2020 [20] (Cohort 1)	69/51	2	-1.08	[-2.74, 0.58]	1.28	0.20	1	0.05	73
Omitting Carubelli et al. 2020 [20] (Cohort 2)	70/50	2	-1.72	[-4.47, 1.03]	1.22	0.22	1	0.0007	91
Omitting Metre et al. 2022 [19] (SEISMiC)	81/39	2	-2.75	[-3.93, -1.57]	4.58	0.00001	1	.49	0

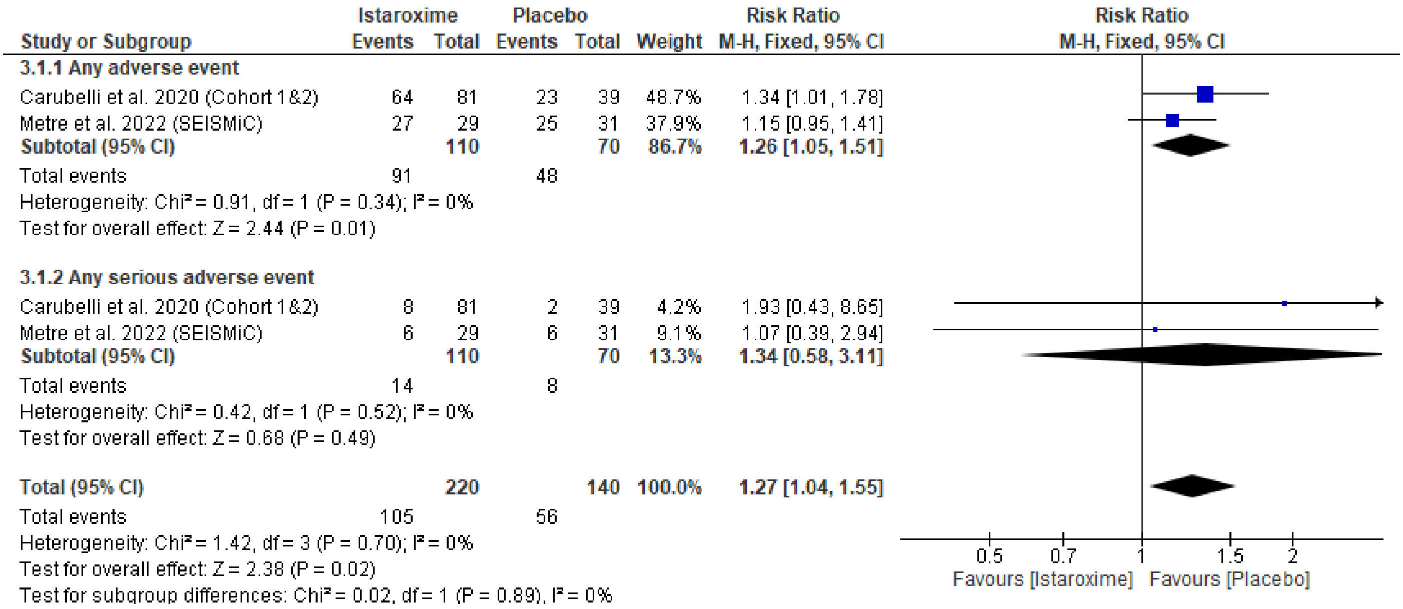

Figure S1. Forest plot of the safety outcomes.
